# Enhanced M1 Macrophage Polarization in Human *Helicobacter pylori*-Associated Atrophic Gastritis and in Vaccinated Mice

**DOI:** 10.1371/journal.pone.0015018

**Published:** 2010-11-23

**Authors:** Marianne Quiding-Järbrink, Sukanya Raghavan, Malin Sundquist

**Affiliations:** Department of Microbiology and Immunology, University of Gothenburg, Gothenburg, Sweden; University of Hyderabad, India

## Abstract

**Background:**

Infection with *Helicobacter pylori* triggers a chronic gastric inflammation that can progress to atrophy and gastric adenocarcinoma. Polarization of macrophages is a characteristic of both cancer and infection, and may promote progression or resolution of disease. However, the role of macrophages and their polarization during *H. pylori* infection has not been well defined.

**Methodology/Principal Findings:**

By using a mouse model of infection and gastric biopsies from 29 individuals, we have analyzed macrophage recruitment and polarization during *H. pylori* infection by flow cytometry and real-time PCR. We found a sequential recruitment of neutrophils, eosinophils and macrophages to the gastric mucosa of infected mice. Gene expression analysis of stomach tissue and sorted macrophages revealed that gastric macrophages were polarized to M1 after *H. pylori* infection, and this process was substantially accelerated by prior vaccination. Human *H. pylori* infection was characterized by a mixed M1/M2 polarization of macrophages. However, in *H. pylori*-associated atrophic gastritis, the expression of inducible nitric oxide synthase was markedly increased compared to uncomplicated gastritis, indicative of an enhanced M1 macrophage polarization in this pre-malignant lesion.

**Conclusions/Significance:**

These results show that vaccination of mice against *H. pylori* amplifies M1 polarization of gastric macrophages, and that a similar enhanced M1 polarization is present in human *H. pylori*-induced atrophic gastritis.

## Introduction

*Helicobacter pylori* colonize the stomach epithelium of more than half of the world's population [1]. The infection is often life-long and triggers a chronic inflammation in the gastric mucosa, which in about 1–2% of infected individuals eventually develops into gastric adenocarcinoma [1]. Development of gastric cancer, in particular the intestinal type, is a multi-step process that progresses over decades through premalignant lesions in the gastric mucosa, such as atrophic gastritis, intestinal metaplasia, and dysplasia [2]. The outcome of the infection depends on the virulence of the infecting *H. pylori* strain, environmental factors such as smoking and diet, and host genetic factors that influence the type and intensity of the inflammatory response [1].

A strong pro-inflammatory response is associated with increased levels of reactive oxygen and nitrogen species in the gastric mucosa [3], which may promote cancer development [4]. For example, mice infected with *H. pylori* for six months have an increased frequency of gastric mutations compared to uninfected mice [5]. In addition, mice that are deficient for the enzyme inducible nitric oxide synthase (iNOS) have a reduced incidence of gastric cancer after *H. pylori* infection and carcinogen challenge compared to normal mice [6]. While iNOS contributes to development of gastric cancer, a high level of the chemokine CCL18 in gastric tumors is associated with prolonged survival of gastric cancer patients [7]. Interestingly, iNOS is produced by classically activated/M1 macrophages whereas CCL18 production is a hallmark for alternatively activated/M2 macrophages [8]. Taken together, these findings suggest that macrophage polarization may have an important role in development of *H. pylori*-associated gastric cancer.

M1 macrophages typically take part in the initial immune response to invading microorganisms and promote T helper (Th) 1 immunity, whereas M2 macrophages are induced during the resolution phase of inflammation and are involved in debris scavenging, tissue remodeling, and promotion of Th2 immunity [8], [9]. Polarization of macrophages is directed by the microenvironment. M1 macrophages are induced by interferon-γ and microbial products such as lipopolysaccharide [9]. On the other hand, M2 macrophages are induced by Th2- or anti-inflammatory cytokines and growth factors, including IL-4, IL-10 and transforming growth factor-β [8], [9].

During *H. pylori* infection, macrophages are recruited to the gastric mucosa, where they contribute to the production of pro-inflammatory cytokines and chemokines [10], [11], [12], [13], [14], [15]. In addition, a recent study showed that liposome-mediated depletion of macrophages reduced gastric pathology in *H. pylori*-infected mice [16]. Despite this, the function of macrophages during in vivo *H. pylori* infection remains relatively poorly defined. The function of macrophages is intimately coupled to their polarization state, which also appears to have a role in development of gastric cancer [6], [7]. Therefore, we have examined macrophage polarization in the gastric mucosa of *H. pylori*-infected mice and humans. We show that vaccination of mice against *H. pylori* speeds and amplifies M1 polarization of gastric macrophages. In addition, the pre-cancerous lesion atrophic gastritis is characterized by an enhanced macrophage M1 polarization in humans.

## Results

### Increased frequency of macrophages, eosinophils and neutrophils in the gastric mucosa after *H. pylori* infection

**Figure 1:**
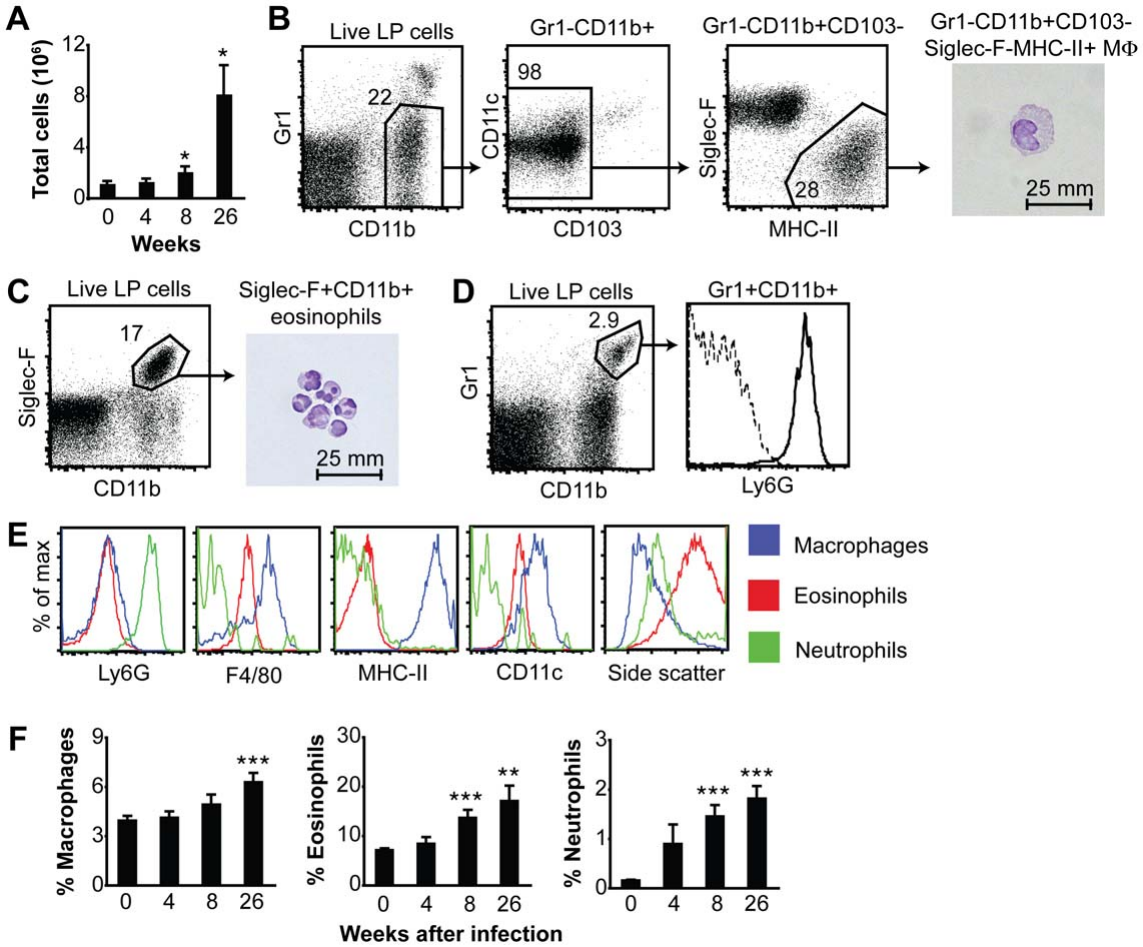
Accumulation of innate cells in the gastric mucosa of *H. pylori*-infected mice. Mice were infected with *H. pylori* SS1 and after 4, 8 and 26 weeks gastric lamina propria cells were isolated from each mouse individually and analyzed by flow cytometry. (A) Total number of cells retrieved from the gastric lamina propria of individual mice at the indicated time points after infection. Data represent mean ± SEM of 5–15 mice. (B) Multiple gating strategy for identification of gastric macrophages, which were defined as Gr1^−^CD11b^+^CD103^−^Siglec-F^−^MHC-II^+^ cells. These cells were sorted, cytospun onto slides, and stained with cresyl violet. LP, lamina propria. MΦ, macrophage. (C) Representative dot plot showing Siglec-F^+^CD11b^+^ eosinophils from an infected mouse. The photomicrograph depicts sorted Siglec-F^+^CD11b^+^ cells prepared as described in *B*. (D) Representative dot plot showing CD11b^+^Gr1^+^ neutrophils from an infected mouse. All CD11b^+^Gr1^+^ cells expressed Ly6G (histogram, thick line). The dotted line represents staining with an isotype control. (E) Surface expression of indicated markers as well as side scatter profile of macrophages (Gr1^−^CD11b^+^CD103^−^Siglec-F^−^MHC-II^+^), eosinophils (Siglec-F^+^CD11b^+^) and neutrophils (CD11b^+^Gr1^+^). (F) Frequencies of macrophages (Gr1^−^CD11b^+^CD103^−^Siglec-F^−^MHC-II^+^), eosinophils (Siglec-F^+^CD11b^+^) and neutrophils (CD11b^+^Gr1^+^) in the gastric lamina propria at the indicated time points after *H. pylori* infection. Data represent means ± SEM of 9–19 individual mice. *, *P*<0.05 **, *P*<0.01 ***, *P*<0.001 compared to uninfected mice via T test.

The recruitment of innate cells to the site of infection is a prerequisite for infectious control. Not only can innate cells, such as macrophages and neutrophils, participate in bacterial killing; they also produce inflammatory mediators, which set the stage for the ensuing immune response. To investigate the accumulation of innate cells in the gastric mucosa during *H. pylori* infection, we infected C57BL/6 mice with the mouse-adapted *H. pylori* Sydney strain 1 (SS1), and after four, eight and 26 weeks we analyzed the gastric inflammatory infiltrate of individual mice by multi-color flow cytometry. The total number of lamina propria cells isolated from the stomach did not change during the first four weeks of infection, but at eight weeks after infection the total number of cells isolated was doubled, and at 26 weeks of infection there was an eight-fold increase in the total number of cells isolated compared to uninfected mice (Fig. 1A). Among the cells being recruited to the stomach were macrophages, eosinophils and neutrophils. Gastric macrophages were identified as cells expressing CD11b and major histocompatibility complex class II (MHC-II), but lacking expression of Gr1 (neutrophil marker), CD103 (expressed by a subset of dendritic cells (DCs)) and sialic acid-binding immunoglobulin-like lectin (Siglec-F, eosinophil marker) (Fig. 1B). These cells expressed the macrophage marker F4/80 (Fig. 1E), and based on cell morphology were confirmed as macrophages (Fig. 1B). The frequency of macrophages in the gastric mucosa remained unchanged after four and eight weeks of *H. pylori* infection (Fig. 1F). However, after 26 weeks the frequency of gastric macrophages was increased compared to uninfected mice (Fig. 1F).

Eosinophils were defined as CD11b^+^Siglec-F^+^ cells (Fig. 1C, [17]). These cells expressed intermediate levels of F4/80 and had a high side scatter profile when analyzed by flow cytometry (Fig. 1E). Cresyl violet staining of sorted CD11b^+^Siglec-F^+^ cells confirmed eosinophil morphology (Fig. 1C). The frequency of eosinophils in the gastric mucosa was doubled after eight weeks and increased further after 26 weeks of infection (Fig. 1F).

Neutrophils were defined as CD11b^+^Gr1^+^ cells (Fig. 1D). Since the Gr1 antibody recognizes both the Ly6C and Ly6G epitope we confirmed that all CD11b^+^Gr1^+^ cells expressed the specific neutrophil marker Ly6G (Fig. 1D and E). The frequency of neutrophils increased 10-fold eight weeks after infection and was further increased after 26 weeks (Fig. 1F). Thus, during *H. pylori* infection there is a sequential accumulation of neutrophils and eosinophils, followed by macrophages in the gastric lamina propria.

### Characterization of gastric DCs

**Figure 2:**
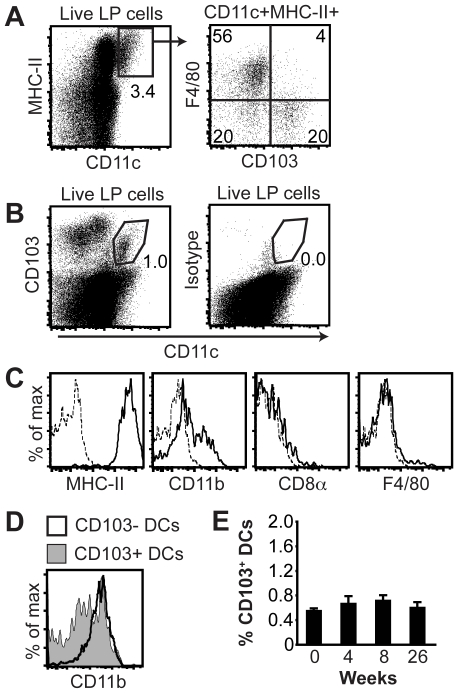
Identification of gastric lamina propria DCs. (A) Flow cytometric analyses of CD11c^+^MHC-II^+^ cells in the gastric lamina propria of individual mice revealed two F4/80^−^ DC populations with differential expression of CD103 (lower quadrants in the F4/80 versus CD103 dot plot). (B) Expression of CD103 and CD11c by gastric lamina propria cells. The gate for CD103^+^ DCs is indicated. Isotype, isotype-matched control antibody for CD103. (C) Expression of MHC-II, CD8α, F4/80 and CD11b by gated CD11c^+^CD103^+^ cells. Dotted lines show staining with isotype controls. (D) Expression of CD11b by CD103^−^ and CD103^+^ DCs. DCs were gated as described in *A*. (E) Frequency of CD103^+^ DCs in the gastric lamina propria at the indicated time points after *H. pylori* infection. Data represents means ± SEM of 7–15 individual mice. LP, lamina propria.

To characterize gastric DCs, we first identified a population of CD11c^+^MHC-II^+^ cells (Fig. 2A). When these cells were further analyzed for expression of F4/80 and the αE integrin chain CD103, half of the CD11c^+^MHC-II^+^ cells were identified as F4/80^+^ macrophages (Fig. 2A). However, among the CD11c^+^MHC-II^+^ cells that lacked expression of F4/80, two populations of putative DCs with differential expression of CD103 could be distinguished (Fig. 2A). Due to the many fluorochromes required we chose to only characterize the gastric CD103*^+^* DCs further, since these cells have been implicated as important antigen presenting cells in mucosal tissues [18]. Gastric CD103^+^ DCs were easily identified by staining for CD11c and CD103 (Fig. 2B). The gastric CD103^+^ DCs expressed high levels of MHC-II, and consisted of a CD11b^low^ and a CD11b^high^ subset (Fig. 2C). In comparison, the gastric CD103^−^ DCs were all CD11b^high^ (Fig. 2D). In addition, the CD103^+^ DCs lacked expression of CD8α and F4/80 (Fig. 2C). However, the frequency of CD103^+^ DCs did not change significantly in the gastric mucosa after infection (Fig. 2E).

### Gastric macrophages and CD103^+^ DCs fail to upregulate costimulatory molecules after *H. pylori* infection

**Figure 3:**
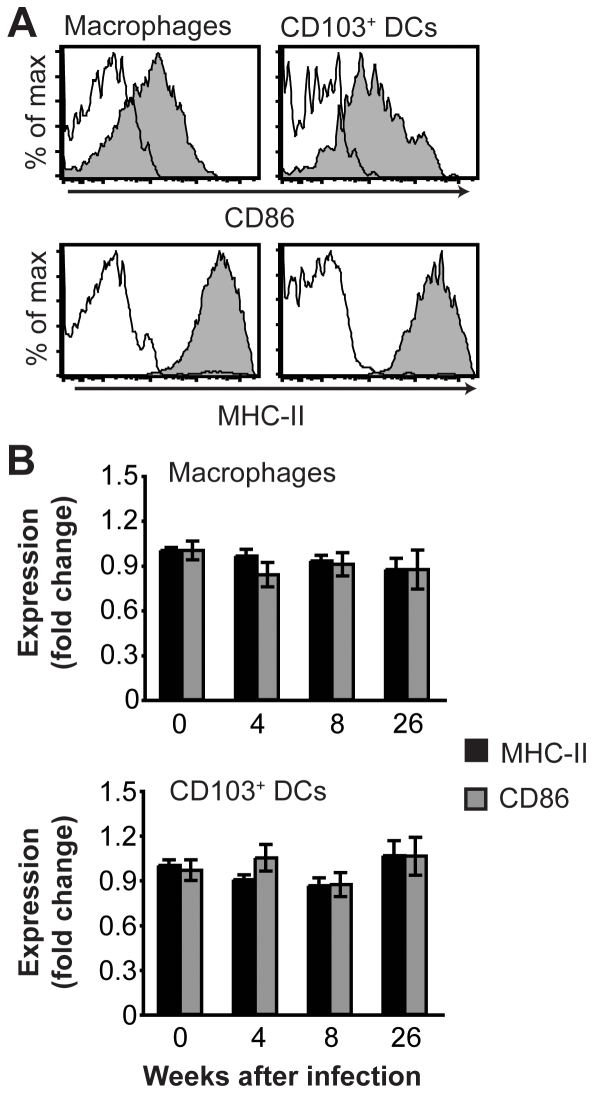
Expression of CD86 and MHC-II by gastric macrophages and DCs. (A) Filled histograms show expression of CD86 and MHC-II by gastric macrophages (Gr1^−^CD11b^+^CD103^−^Siglec-F^−^MHC-II^+^) and CD103^+^ DCs (CD11c^+^CD103^+^) from a naïve mouse. Open histograms show staining with isotype controls. (B) Expression of CD86 and MHC-II by gastric macrophages and CD103^+^ DCs at the indicated time points after *H. pylori* infection as determined by flow cytometry. The geometric mean fluorescence intensity of CD86 and MHC-II was normalized to the level in age-matched uninfected mice. Data represent mean ± SEM of 10–16 individual mice.

In order to investigate the possible effects of *H. pylori* infection on the expression of costimulatory molecules and MHC-II by gastric macrophages and CD103^+^ DCs, the expression of these markers was analyzed by flow cytometry after four, eight and 26 weeks of infection. In the steady state, macrophages and CD103^+^ DCs in the gastric lamina propria expressed similar levels of the costimulatory molecule CD86 as well as MHC-II (Fig. 3A). Surprisingly, the expression of CD86 and MHC-II by either gastric macrophages or CD103^+^ DCs was not increased after infection with *H. pylori* relative to age-matched naïve mice analyzed in parallel (Fig. 3B). Thus, macrophages and CD103^+^ DCs in the gastric lamina propria fail to upregulate CD86 and MHC-II after *H. pylori* infection, despite the ongoing inflammatory response.

### M1 polarization of gastric macrophages during *H. pylori* infection

**Figure 4:**
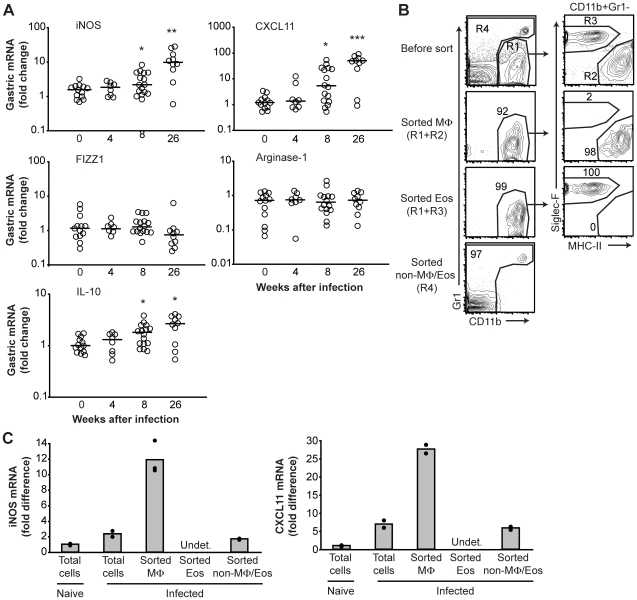
M1 polarization of gastric macrophages after *H. pylori* infection. (A) Expression of genes associated with macrophage polarization in the gastric mucosa of naïve and infected mice was determined by real-time PCR. Symbols represent individual mice, and horizontal bars indicate the medians. *, *P*<0.05 **, *P*<0.01 compared to the expression level in uninfected mice via Mann-Whitney U test. (B) Sorting of gastric lamina propria macrophages (Gate R1 and R2, CD11b^+^Gr1^−^Siglec-F^−^MHC-II^+^), eosinophils (Gate R1 and R3, CD11b^+^Gr1^−^Siglec-F^+^MHC-II^−^) and remaining cells after gating out macrophages and eosinophils (Gate R4, CD11b^−^ and CD11b^+^Gr1^+^) from pooled (n = 3) mice infected for 26 weeks. Contour plots show expression of CD11b versus Gr1 on gated live cells, and expression of Siglec-F versus MHC-II on gated CD11b^+^Gr1^−^ cells before and after sorting. The gates for sorting (R1–R4) are shown in the top contour plots. Numbers by gates depict cell frequencies. Note that in this experiment the few CD103^+^ DCs that fall into the macrophage gate (∼2%, see Fig. 1A) were not gated out. (C) Real-time PCR analysis of iNOS and CXCL11 mRNA expression by total gastric lamina propria cells pooled from naïve (n = 3) or infected (n = 3, 26 weeks) mice before sorting as well as expression by sorted macrophages (Gate R1 and R2, CD11b^+^Gr1^−^Siglec-F^−^MHC-II^+^, 9×10^5^ cells), eosinophils (Gate R1 and R3, CD11b^+^Gr1^−^Siglec-F^+^MHC-II^−^, 6×10^5^ cells) and remaining cells after gating out macrophages and eosinophils (Gate R4, CD11b^−^ and CD11b^+^Gr1^+^, 3×10^6^ cells) from infected mice. Bars are the mean of two to three replicates, and dots represent each replicate. Undet., undetected. MΦ, macrophages. Eos, eosinophils.

Since our results suggested that the gastric macrophages might not be fully activated during *H. pylori* infection, we characterized these cells further by investigating their M1/M2 polarization. To determine macrophage polarization during *H. pylori* infection, we used real-time PCR to measure the expression of genes associated with M1 or M2 polarization of macrophages in the gastric tissue [8], [9]. We also measured the expression of IL-10, which can be produced by regulatory macrophages [9]. None of the markers analyzed were differentially expressed four weeks after *H. pylori* infection relative to naïve mice (Fig. 4A). At eight weeks after infection the expression of the M1 markers iNOS and CXCL11 was significantly increased, and these markers were further upregulated at 26 weeks of infection (Fig. 4A). In addition, the expression of IL-10 was upregulated at eight and 26 weeks of infection relative to naïve mice (Fig. 4A). In contrast, the M2 markers found in inflammatory zone 1 (FIZZ1) and arginase-1 were not differentially expressed in the stomach at four, eight or 26 weeks of infection compared to naïve mice (Fig. 4A).

To identify the source of iNOS and CXCL11 in the gastric mucosa, macrophages (CD11b^+^Gr1^−^Siglec-F^−^MHC-II^+^), eosinophils (CD11b^+^Gr1^−^Siglec-F^+^MHC-II^−^) and the remaining cells after gating out macrophages and eosinophils (CD11b^−^ and CD11b^+^Gr1^+^) were sorted from pooled gastric lamina propria cells of mice infected with *H. pylori* for 26 weeks (Fig. 4B). The mRNA expression of iNOS and CXCL11 in sorted cell populations from infected mice as well as in total gastric lamina propria cells from both naïve and infected mice was then determined by real-time PCR. A sufficient number of sorted macrophages could not be obtained from naïve mice for reliable analysis of mRNA expression. Gastric macrophages expressed the highest level of iNOS and CXCL11 compared to the other sorted cell populations and total gastric lamina propria cells before sorting (Fig. 4C). Taken together, these results show that gastric macrophages are polarized to M1 during *H. pylori* infection.

### Vaccination against *H. pylori* amplifies macrophage M1 polarization

**Figure 5:**
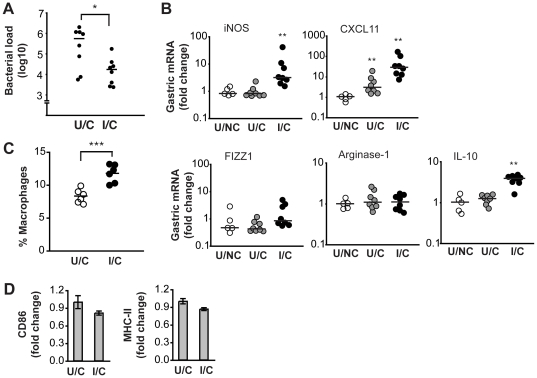
M1 polarization of gastric macrophages in immunized mice following challenge with *H. pylori*. Mice were immunized with *H. pylori* lysate and cholera toxin and challenged with *H. pylori* SS1 (I/C: immunized/challenged). A second group of mice was infected with SS1 without prior immunization (U/C: unimmunized/challenged) and a third group was left untreated (U/NC: unimmunized/non-challenged). (A) Number of bacteria cultured from the stomach of U/C and I/C mice four weeks after challenge. Horizontal bars indicate the median. *, *P*<0.05 via Mann-Whitney U test. (B) Four weeks after challenge the expression of genes associated with macrophage polarization was determined in the gastric mucosa of the mice shown in *A* by real-time PCR. Symbols represent individual mice, and horizontal bars indicate the medians. **, *P*<0.01 compared to the expression level in U/NC mice via Mann-Whitney U test. (C) In separate experiments, the frequency of gastric macrophages (Gr1^−^CD11b^+^CD103^−^Siglec-F^−^MHC-II^+^) in U/C and I/C mice was determined by flow cytometry three weeks after *H. pylori* infection. Each symbol represents individual mice, and horizontal bars indicate the mean. ***, *P*<0.001 via T test. (D) Flow cytometric analysis of CD86 and MHC-II expression by gastric macrophages (Gr1^−^CD11b^+^CD103^−^Siglec-F^−^MHC-II^+^) from the U/C and I/C mice shown in *C*. The geometric mean fluorescence intensity was normalized to the level in U/C mice. Data are presented as mean ± SEM.

Protective immunization against *H. pylori* is generally associated with the rapid development of a gastric Th1 response [19]. Since the Th1 cytokine interferon-γ induces macrophage M1 polarization [9], we examined whether vaccination could influence macrophage polarization during *H. pylori* infection. To this end, mice were immunized sublingually with *H. pylori* lysate and cholera toxin adjuvant, and subsequently challenged with *H. pylori* SS1. This immunization regimen led to a significant reduction in bacterial load in the stomach four weeks after challenge (Fig. 5A). At the same time, the expression of M1 and M2 markers in the stomach was analyzed by real-time PCR. In unimmunized mice, only the expression of CXCL11 was significantly upregulated four weeks after infection compared to naïve mice (Fig. 5B). In contrast, immunized mice showed a great upregulation of both the M1 markers iNOS and CXCL11 whereas the expression of the M2 markers FIZZ1 and arginase-1 was not changed, four weeks after challenge (Fig. 5B). The increased expression of iNOS and CXCL11 in immunized/challenged mice was not due to the immunization alone, since immunized but non-challenged mice did not change the expression of any of the analyzed M1 or M2 markers compared to completely untreated mice (data not shown).

In addition, we analyzed the frequency of macrophages in the gastric mucosa of immunized and challenged mice by flow cytometry. Relative to infected-only mice, immunized mice had a significantly increased frequency of gastric macrophages three weeks after challenge (Fig. 5C). However, despite that macrophages were recruited to the gastric mucosa of immunized and challenged mice, the macrophages did not upregulate the expression of CD86 or MHC-II relative to infected-only mice (Fig. 5D). These results show that after successful vaccination with *H. pylori* lysate and cholera toxin, macrophages accumulate in the gastric mucosa and are rapidly polarized to M1 after infection.

### Augmentation of macrophage M1 polarization in human atrophic gastritis

**Figure 6:**
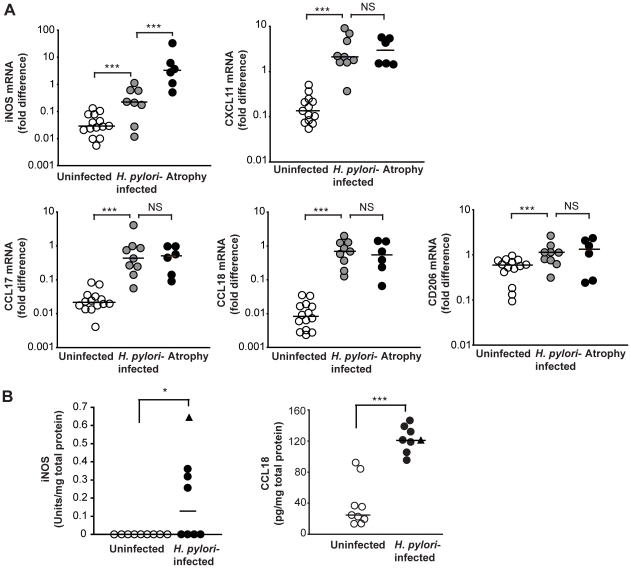
Increased M1 macrophage polarization in human atrophic gastritis. (A) The expression of genes associated with human M1 (iNOS, CXCL11) or M2 (CCL17, CCL18, CD206) macrophage polarization was determined in the gastric mucosa of uninfected and *H. pylori*-infected individuals with uncomplicated or atrophic gastritis by real-time PCR. Symbols represent individual values and horizontal bars indicate the medians. (B) Concentration of CCL18 and iNOS in protein extracts of gastric biopsy specimens from uninfected and *H. pylori*-infected individuals. Filled circles represent individuals with uncomplicated *H. pylori*-associated gastritis and the triangle represents a single individual with atrophic gastritis. Horizontal bars indicate the medians. *, *P*<0.05 ***, *P*<0.001 via Mann-Whitney U test.

We next investigated the role of *H. pylori* infection and atrophic gastritis for macrophage polarization in the human gastric mucosa. To this end, the expression of human M1 (iNOS, CXCL11) and M2 markers (CCL17, CCL18, CD206) was measured in biopsies from antrum by real-time PCR. *H. pylori*-infected individuals with uncomplicated gastritis showed a significantly elevated expression of mRNA for both M1 (iNOS, 8-fold; CXCL11, 20-fold) and M2 markers (CCL17, 30-fold; CCL18, 70-fold, CD206, 2-fold) compared to uninfected volunteers (Fig. 6A). However, individuals with atrophic gastritis (4/6 had intestinal metaplasia in addition to atrophy) expressed even higher levels of iNOS mRNA compared to those with uncomplicated gastritis (20-fold), whereas CXCL11 and markers of M2 polarization were similarly expressed (Fig. 6A). Indeed, the expression of iNOS was 180-fold increased in individuals with atrophic gastritis compared to uninfected controls (Fig. 6A), indicating an enhanced M1 polarization of gastric macrophages in patients with atrophic gastritis.

To investigate whether the increased mRNA expression of M1 and M2 markers also translates into increased protein levels, total proteins were extracted from antral biopsy specimens and the concentration of iNOS and CCL18 was determined by ELISA. Gastric biopsies from individuals with atrophic gastritis was only available for protein extraction from one volunteer, which is differentially indicated in Figure 6B. Half of the *H. pylori*-infected individuals had detectable levels of iNOS protein in the antrum whereas the concentration of iNOS was below the detection limit in all uninfected individuals (Fig. 6B). The expression of iNOS mRNA and iNOS protein was significantly correlated (R^2^ = 0.88, *P*<0.01) indicating that the mRNA analysis reflects protein expression, even when the protein levels are low. The concentration of the M2 marker CCL18 was increased in the gastric tissue from *H. pylori*-infected individuals compared to uninfected controls (Fig. 6B). Moreover, the concentration of CCL18 protein was significantly correlated with the expression of CCL18 mRNA (R^2^ = 0.754, *P*<0.01).

Taken together, these findings indicate the presence of both M1 and M2 macrophages in the gastric mucosa of *H. pylori*-infected individuals. Furthermore, atrophic gastritis is associated with a strong amplification of iNOS expression in the gastric mucosa, indicating an enhanced M1 polarization of macrophages.

## Discussion

In this study, we have investigated the polarization of gastric macrophages during chronic *H. pylori* infection. We show that *H. pylori*-infected individuals express mRNA in the gastric mucosa indicative of a mixed M1/M2 polarization of macrophages, and this was further confirmed at the protein level. However, in *H. pylori*-induced atrophic gastritis there was a marked elevation in the expression of iNOS compared to uncomplicated gastritis. Atrophic gastritis confers an increased risk of developing gastric cancer relative to uncomplicated *H. pylori*-associated gastritis [20]. The increased expression of iNOS in atrophic gastritis may contribute to gastric cancer development via production of reactive nitrogen species, which can promote carcinogenesis by induction of DNA damage, disruption of DNA repair, post-translational modification of proteins, and p53 mutations [4]. Indeed, iNOS-deficient mice have a reduced incidence of gastric adenocarcinoma after *H. pylori* infection and challenge with a chemical carcinogen compared to normal mice [6]. Also, polymorphisms in the promoter region of iNOS, leading to a higher transcriptional activity, correlate with a higher incidence of the intestinal type of gastric cancer in Japanese women [21].

In contrast to human *H. pylori* infection, SS1-infected C57BL/6 mice showed a gene expression profile in the gastric mucosa indicative of macrophage M1 polarization. Gene expression analysis of sorted macrophages isolated from the gastric mucosa of infected mice confirmed that expression of the M1 markers iNOS and CXCL11 was enriched in the sorted macrophage population. Furthermore, the M1 polarization of gastric macrophages was substantially accelerated by prior vaccination. Already four weeks after challenge, immunized mice upregulated the expression of iNOS and CXCL11 to a similar level as that seen after 26 weeks in infected-only mice. Studies of iNOS-deficient mice have shown that iNOS promotes development of atrophy and cancer in the gastric mucosa during *Helicobacter* infection [6], [22]. Moreover, clearance of *H. pylori* after vaccination occurs independently of iNOS [23]. Thus, iNOS appears to contribute to host pathology rather than protection during infection with *H. pylori*. Therefore, if the enhanced production of iNOS in the gastric mucosa is maintained after vaccination, it may be an unwanted side effect that can augment the severity of *H. pylori*-induced inflammation and malignancy, unless sterile immunity is achieved. However, the relative contribution of iNOS to host pathology versus protection during different stages of *H. pylori* infection warrants further investigation.

We observed a sequential recruitment of innate cells to the gastric mucosa of SS1-infected mice, with the macrophages accumulating rather late during the course of infection (26 weeks). In contrast, the frequencies of gastric neutrophils and eosinophils in the gastric mucosa increased eight weeks after infection and remained elevated at 26 weeks. The accumulation of macrophages occurred much faster in vaccinated mice, in which case the frequency of gastric macrophages was increased already three weeks after challenge. Previously, neutrophils have been shown to be recruited to the gastric mucosa of SS1-infected mice in two phases, one early phase peaking 1–2 days after infection and one late phase beginning at 2–3 weeks after infection [24]. A recruitment pattern, similar to that of neutrophils, was also described for gastric macrophages [24]. However, the macrophages were defined as CD11b^+^Gr1^−^ cells [24], a cell population that in our hands primarily consists of eosinophils (see Fig. 1 and 2). Asim et al. defined macrophages as CD11b^+^F4/80^+^ cells, and observed an early peak in macrophage number in the gastric mucosa 1–2 days after infection with *H. pylori* [15]. In addition, the number of macrophages was increased again at 60 and 120 days after infection relative to naïve mice [15]. We extend these results by showing that the recruitment of these innate cell populations is maintained up until 26 weeks after infection. In addition, we describe the accumulation of eosinophils in the gastric mucosa, a cell population that by far outnumber the gastric macrophages and neutrophils (Fig. 1). Indeed, the role of eosinophils in *H. pylori*-induced gastritis should be investigated further. Eosinophils and macrophages share expression of several markers, including CD11b and F4/80 (Fig. 1, [25]). Therefore, multiple markers need to be analyzed simultaneously in order to distinguish these innate cell populations.

We were able to identify two populations of DCs in the gastric mucosa of mice. Both subsets expressed high levels of CD11c and MHC-II, lacked expression of the macrophage marker F4/80, but differed with regards to expression of CD103. The frequency of gastric CD103^+^ DCs did not change after four, eight or 26 weeks of *H. pylori* infection. In contrast to our study, a recent study by Kao et al. could not detect CD103^+^ DCs in the gastric mucosa of uninfected mice [26]. Rather, the CD103^+^ DCs emerged 24 hr after *H. pylori* infection. Since the times of analysis differ between that study and our experiments, it is hard to directly compare the results.

Despite that M1 macrophages typically upregulate MHC-II and costimulatory molecules, we could not detect an increased expression of MHC-II or CD86 on gastric macrophages or CD103^+^ DCs from either unimmunized or immunized mice after infection. In contrast, in vitro incubation of *H. pylori* with human monocytes [27], human monocyte-derived DCs [28], [29], [30], [31], murine bone marrow-derived DCs [32], [33], or human primary gastric DCs [34], induces upregulation of costimulatory molecules and MHC-II. Therefore the failure to upregulate MHC-II and CD86 on gastric macrophages and CD103^+^ DCs in chronic *H. pylori* infection may be caused by the inflammatory milieu and not by the bacteria per se. Indeed, the interaction between DCs and *H. pylori* in vitro does not necessarily reflect what happens in vivo, where the local microenvironment at the site of antigen acquisition and antigen presentation as well as the DC subset involved in antigen presentation of *H. pylori*, will influence the response. For example, regulatory T cells, which are prevalent in the *H. pylori*-infected gastric mucosa [35], [36], can prevent the upregulation of costimulatory molecules and MHC-II on DCs [37]. In addition, IL-10 can prevent the upregulation of costimulatory molecules and MHC-II on macrophages and DCs [38].

During acute inflammatory responses, macrophages are typically polarized to M1 and exert potent anti-microbial effects. For example, infection with *Salmonella typhimurium* or *Listeria monocytogenes* induces M1 polarization of macrophages, which is required for control of the infection [39]. Resolution of inflammation is characterized by a shift in macrophage polarization to M2, which promotes tissue healing. Tuberculosis patients display an increased production of Th2 cytokines as the infection progresses [40], [41], which can induce M2 polarization of macrophages. In mice, M1 polarization of macrophages constitutes the early response to *Mycobacterium tuberculosis*, whereas alveolar macrophages are polarized to M2 during late stage infection [42]. However, the shift in macrophage polarization induced by chronic infections often results in a reduced capacity of the macrophages to kill the invading bacteria [39], [43]. On the other hand, chronic inflammation with persistent M1 macrophage polarization is associated with an increased risk of cancer development [4].

Do macrophages contribute to host defense against *H. pylori*? Unlike neutrophils, macrophages are not frequently seen in the gastric lumen after translocation across the epithelium [44]. Since *H. pylori* preferentially reside in the gastric mucus layer or are attached to gastric epithelial cells, macrophages may not come in direct contact with whole bacteria. Indeed, depletion of macrophages by drug-loaded liposomes had no effect on *H. pylori* colonization [16], suggesting that macrophages may not directly contribute to host defense against *H. pylori*. In contrast, macrophages may promote gastric pathology. For example, liposome-mediated depletion of macrophages ameliorated the gastritis induced by *H. pylori* infection [16]. In addition, selective deletion of I-κB-kinase β in myeloid cells, which prevents activation of NF-κB in these cells, inhibited the development of gastric atrophy after *H. felis* infection [45]. Thus, macrophages do not appear to contribute to *Helicobacter* clearance, but may rather promote gastric pathology.

In conclusion, this study shows that vaccination of mice against *H. pylori* amplifies M1 polarization of gastric macrophages. A similar phenomenon is seen in human atrophic gastritis where the mixed M1/M2 polarization present in uncomplicated gastritis is replaced by an M1-dominated polarization. This may induce a tumor-promoting inflammation, and shifting macrophage polarization from M1 to M2 could therefore represent a therapeutic target in chronic *H. pylori* infection.

## Materials and Methods

### Ethics statement

The study was approved by the government animal ethics committee (Göteborgs djurförsöksetiska nämnd, 328/2008 and 254/2009). The regional human ethics committee of Västra Götaland County in Sweden (706/03 and 85/06) approved the study, and written informed consent was obtained from all participants. This is the only authority giving ethical permission for research on humans in Sweden, and it is not directly associated to hospitals or universities.

### Mice

Female C57BL/6 mice were purchased from Charles River Laboratories (Sulzfeld, Germany), or in the case of the vaccination experiments from Taconic (Ejby, Denmark). Mice were infected at an age of 8–12 weeks.

### Bacteria and infection of mice

*H. pylori* SS1 was grown on Columbia ISO agar plates for 2 days at 37°C, after which they were transferred to Brucella broth supplemented with 5% fetal calf serum (FCS) and antibiotics (Vancomycin, 10 µg/ml; Polymyxin B, 20 U/ml; Trimethoprim, 5 µg/ml) and incubated shaking over night at 37°C under microaerophilic conditions. Before infection, the motility of the bacteria was confirmed by microscopy. The concentration of bacteria was estimated spectrophotometrically. Mice received 3×10^8^ colony-forming units of SS1 intragastrically.

### Sublingual immunization

Mice were given four 10 µl doses of 500 µg *H. pylori* lysate (prepared from strain Hel305 as previously described [46]) combined with 10 µg cholera toxin (List Biological Laboratories Inc., Madison, NJ) sublingually at 1-week intervals [47], [48]. Two weeks after the last immunization, the mice were challenged intragastrically with 3×10^8^ colony-forming units of *H. pylori* SS1.

### Assessment of bacterial colonization

For quantitative assessment of bacterial colonization in immunization experiments, one half of the stomach was gently washed with PBS and homogenized using an Ultra Turrax homogenizer (IKA Laboratory Technology, Staufen, Germany). Serial dilutions of the homogenate were plated on Skirrow agar plates. When gastric lamina propria cells were isolated from the whole stomach a quantitative estimation of the gastric bacterial load could not be performed. In this case, the stomach, which was cut along the greater curvature and washed in PBS, was gently streaked on Skirrow agar plates. The presence of *H. pylori* colonies was confirmed by a urease test. In this way we could confirm *H. pylori* colonization and still use the whole stomach for cell isolation.

### Isolation of gastric lamina propria cells

The glandular stomach was cut into 5 mm pieces and incubated a total of three times with 2 mM EDTA in Hanks' balanced salt solution (HBSS) (Invitrogen) supplemented with 2% FCS for 15 min at 37°C, followed by vigorous shaking for 15 s and filtration to remove epithelial cells. The remaining tissue was incubated with 100 U/ml collagenase type VIII (Sigma-Aldrich, St Louis, MO) in RPMI 1640 containing 10% FCS and 10 mM HEPES for 60 min at 37°C. The released lamina propria cells were collected by filtration through a nylon mesh, and the remaining tissue was subjected to a second round of collagenase digestion. Leukocytes were further enriched on a 40∶70 Percoll gradient after centrifugation at 600 g for 20 min.

### Flow cytometry

To block unspecific binding, single-cell suspensions were incubated with anti-Fcγ receptor II/III monoclonal antibody (2.4G2, own production). Then cells were stained with the following monoclonal antibodies: anti-CD11b-PE or -allophycocyanin-Cy7 (M1/70), Gr1-PE-Cy7 (RB6-8C5), Siglec-F-PE (E50-2440), CD103 biotinylated (M290), Ly6G-FITC (1A8), CD11c-FITC (HL3), CD86-PE (GL1), MHC-II-PE (M5/114.15.2), and CD8α-allophycocyanin (53-6.7) (BD Biosciences, San Jose, CA), MHC-II-AlexaFluor 700 (M5/114.15.2) (Biolegend, San Diego, CA), and F4/80-PE (CI:A3-1) (Caltag Laboratories, Buckingham, UK). 7-Aminoactinomycin D (7AAD, Sigma-Aldrich) was included for dead cell exclusion. For biotinylated antibodies streptavidin-allophycocyanin was used as a second-step reagent (BD Biosciences). Samples were acquired on an LSR-II flow cytometer (BD Biosciences) and analyzed using FlowJo software (Tree star, Ashland, OR).

### Cell sorting

For morphological studies, gastric eosinophils (7AAD^−^, CD11b^+^, Siglec-F^+^) and macrophages (7AAD^−^, CD11b^+^, Gr1^−^, CD103^−^, Siglec-F^−^, MHC-II^+^) were sorted with a FACSAria (BD Biosciences). The purity of sorted cells was >96%. Cytospin preparations of purified eosinophils and macrophages were stained with cresyl violet.

For gene expression analysis, gastric macrophages (7AAD^−^, CD11b^+^, Gr1^−^, Siglec-F^−^, MHC-II^+^; 9×10^5^ cells), eosinophils (7AAD^−^, CD11b^+^, Gr1^−^, Siglec-F^+^, MHC-II^−^; 6×10^5^ cells), and the remaining cells after gating out macrophages and eosinophils (7AAD^−^, CD11b^−^ and 7AAD^−^, CD11b^+^, Gr1^+^; 3×10^6^ cells) were sorted to >92% purity. Total RNA was isolated using the Qiagen RNeasy mini kit and transcribed into complementary DNA using the Omniscript kit (Qiagen). Real-time PCR was performed as described below.

### Volunteers and specimen collection

Ten *H. pylori*-infected volunteers and nine uninfected individuals were recruited among healthy blood donors at Sahlgrenska University hospital after serological screening. In addition, the study included five *H. pylori*-infected patients with atrophic gastritis and five uninfected individuals, who underwent endoscopy at Sahlgrenska University hospital because of abdominal complaints. *H. pylori* infection was confirmed or excluded by culture on Skirrow agar plates as well as by serology [49] and pathology reports. Three antrum biopsy specimens were put in RNA Later (Ambion, Austin, TX) and then stored at −70°C until RNA isolation. An additional three antral biopsies were snap frozen in liquid nitrogen and stored at −70°C for subsequent protein extraction. One biopsy specimen from antrum (all individuals) and corpus (volunteers recruited at the hospital) was examined by an experienced histopathologist, and the grade of gastritis, including presence of atrophic gastritis and intestinal metaplasia, was determined according to the updated Sydney system [50]. In total, the study included nine individuals with uncomplicated *H. pylori*-associated gastritis (mean age 47 years, range 32–67 years, five females), six *H. pylori*-infected individuals with atrophic gastritis (mean age 74 years, range 63–81 years, three females) and 14 uninfected individuals (mean age 49 years, range 27–75 years, seven females).

### Real-time RT-PCR

Human antral biopsies as well as longitudinal strips from the greater curvature of the mouse stomach were homogenized using a TissueLyserII (Qiagen, Valencia, CA). Total RNA was extracted using the Qiagen RNeasy mini kit and transcribed into complementary DNA using Oligo(dT) and the Omniscript kit (Qiagen). Real-time PCR was performed with TaqMan gene expression assays and the TaqMan Universal Master Mix from Applied Biosystems (Foster City, CA). Reactions were run in duplicate using standard amplification conditions for the 7500 real-time PCR system (Applied Biosystems). Relative expression levels were determined by the ΔΔCt method using hypoxanthine phosphoribosyltransferase 1 (HPRT1) and β-actin as reference genes for human and mouse expression assays, respectively. Data are expressed as change in mRNA expression relative to a calibrator sample. For human samples, a tonsil specimen was used as calibrator, whereas a gastric sample from one of the naïve mice was used as calibrator in mouse assays. The following primer sets from Applied Biosystems were used for human genes: HPRT1 (Hs99999909-m1), CCL17 (Hs00171074-m1), CCL18 (Hs00268113-m1), CD206 (Hs00267207-m1), iNOS (Hs00167257-m1), CXCL11 (Hs00171138-m1). The primer sets used for mouse genes were as follows: β-actin (Mm01205647-g1), FIZZ1 (Mm00445109-m1), arginase-1 (Mm00475988-m1), IL-10 (Mm01288386-m1), iNOS (Mm01309897-m1), CXCL11 (Mm00444662-m1).

### Detection of CCL18 and iNOS in human gastric protein extracts

Three antral biopsies from each individual were incubated in 600 µl PBS containing saponin, soybean trypsin inhibitor, Pefabloc, and bovine serum albumin overnight at 4°C as previously described [51]. The concentration of CCL18 and iNOS in the protein extracts was determined using a Duoset ELISA for CCL18 (R&D systems, Abingdon, United Kingdom) and a Quantikine ELISA for iNOS (R&D systems) according to the manufacturer's instructions. The extracts were desalted on Zeba Micro Desalt spin columns (Thermo Scientific, Rockford, IL) and total protein concentrations were measured using the BCA protein assay (Thermo Scientific). The concentration of CCL18 and iNOS was normalized to the total protein content of the extracts.

### Statistics

Statistical analysis was performed using SPSS 17.0 software. Means were compared using an independent samples T test and Levene's test for equality of variances. Non-parametric data was analyzed using a two-tailed Mann-Whitney U test. Correlation was evaluated using a two-tailed Pearson test. Values of *P*<0.05 were considered significant.
